# HPV associated tumor cells control tumor microenvironment and leukocytosis in experimental models

**DOI:** 10.1002/iid3.21

**Published:** 2014-05-18

**Authors:** Simone Cardozo Stone, Renata Ariza Marques Rossetti, Aleida Maria Lima, Ana Paula Lepique

**Affiliations:** Institute of Biomedical Sciences, Department of Immunology, University of Sao PauloAv. Prof. Lineu Prestes, 1730, Room 136, 05508-900, Sao Paulo, SP, Brazil

**Keywords:** cervical cancer, cytokines, inflammation, human papillomavirus, leukocytosis

## Abstract

Human papillomavirus (HPV) is the main etiological factor for cervical cancer development. HPV is also associated with other anogenital and oropharyngeal tumors. HPV associated tumors are frequent and constitute a public health problem, mainly in developing countries. Therapy against such tumors is usually excisional, causing iatrogenic morbidity. Therefore, development of strategies for new therapies is desirable. The tumor microenvironment is essential for tumor growth, where inflammation is an important component, displaying a central role in tumor progression. Inflammation may be a causal agent, suppressor of anti-tumor T cell responses, or may have a role in angiogenesis, drug resistance, and metastasis. The aim of this work was to investigate the role of HPV transformed cells in the tumor microenvironment and tumor effects on myeloid populations in lymphoid organs in the host. We used experimental models, where we injected cervical cancer derived cell lines in immunodeficient mice, comparing HPV positive, SiHa, and HeLa cells (HPV 16 and HPV18, respectively), with HPV negative cell line, C33A. Our data shows that HPV positive cell lines were more efficient than the HPV negative cell line in leukocyte recruitment to the tumor microenvironment and increase in myeloid cell proliferation in the bone marrow and spleen. We also observed that HPV positive cells lines expressed significantly higher levels of IL-6 and IL-8, while C33A expressed significantly higher levels of IL-16 and IL-17. Finally, in spite of cytokine secretion by tumor cells, leukocytes infiltrating SiHa and HeLa tumors displayed almost negligible STAT3 and no NFκB phosphorylation. Only the inflammatory infiltrate of C33A tumors had NFκB and STAT3 activated isoforms. Our results indicate that, although from the same anatomical site, the uterine cervix, these cell lines display important differences regarding inflammation. These results are important for the design of immunotherapies against cervical cancer, and possibly against HPV associated tumors in other anatomical sites.

## Introduction

Persistent infection with high oncogenic risk human papillomavirus (HPV) is the main cause for a percentage of anogenital and oropharyngeal tumors and virtually all cervical tumors [1,2]. Upon HPV infection, tumor development involves persistence of the virus in the basal epithelial cell layer and viral DNA integration into the host cell's genome in a way that E6 and E7 oncogenes are constitutively expressed, causing cell immortalization [3,4]. Through time, immortalized cells acquire more mutations or HPV oncoproteins cause further disruptions leading to cell transformation [5]. The expression of HPV oncoproteins E6 and E7 is essential for the maintenance of the transformed phenotype of HPV associated tumor cells, as shown by senescence induction when E6 and E7 expression was inhibited in cervical cancer derived cells [6,7]. Throughout tumor development, immune evasion mechanisms are important [8]. HPV infected cells, immortalized or transformed, display several immune evasion mechanisms, among them non-lytic viral cycle, decrease in the expression of antigen presentation machinery and inhibition of Interferon type I triggered pathways [8]. The host, however, is capable of mounting efficient immune responses against tumor antigens, so that most of the infected women eliminate the virus and cervical precursor lesions [9,10]. Tumor development occurs when immune evasion mechanisms are more efficient than immune responses, allowing tumor cells to grow, at least partially unchecked by the immune system [8].

In cancer development, inflammation is a two edged sword: while essential for triggering of anti-tumor responses, it can also promote tumor progression and growth. Chronic inflammation, through production of oxygen and nitrogen reactive species, can promote mutations and chronic tissue lesion [11,12]. It may also be responsible for chronic secretion of angiogenic and mitogenic factors that may favor tumor growth [13]. Finally, in grown tumors, suppressor cytokines, as TGFβ and IL-10, and metabolites, influence the phenotype of infiltrating inflammatory cells inducing suppressive phenotype to generate M2 macrophages, N2 neutrophils, myeloderived suppressor cells (MDSC) and regulatory T cells [14,15].

Among lesions generated by HPV, cervical cancer is the best-studied one. Recently, publications regarding HPV associated lesions or tumors and inflammation are increasing. Although most of the published data points to an anti-inflammatory response, there is evidence of oxidative stress as well as Th2 responses playing a role in cervical cancer development [16,17]. There is data regarding the inflammatory infiltrate in patients and experimental models showing CD8 infiltration high grade lesions [18], regulatory CD4 T cells, CD4 and CD8 T cells infiltrating tumors [19], as well as myeloid cells, both macrophages and dendritic cells with potentially suppressor phenotype in cancer [20]. Moreover, some studies have shown an increase in the frequency of macrophages in proportion to tumor grade [21,20]. In experimental models, it has been shown that macrophages are important for angiogenesis and suppression of T cell anti-tumor responses [22,23]. Systemic effects of HPV associated tumors usually comprise alterations in circulating cytokine concentration or frequency of cells in the peripheral blood of patients. Recent data has shown that patients with advanced cervical cancer may suffer of myeloid cell leukocytosis, which represents a poor prognostic in patients with recurrent disease [24,25]. In experimental models, HPV associated tumors induce suppressive responses in secondary lymphoid organs [23,26], where myeloid derived suppressor cells inhibit T cell responses in a MHC-I dependent manner [26]. Through all this piece of information, one point seems to be clear, developed tumors are infiltrated by myeloid cells with suppressor phenotype, which also seem to be more frequent in peripheral lymphoid organs.

Using experimental models, we have shown that depleting tumor associated macrophages or neutralizing cytokines [23,27], it is possible to interfere with tumor growth, creating opportunities, where conventional therapies as chemotherapy and radiotherapy, or even other types of immunotherapy, may display more efficient or complete responses against cancer. Therefore, the aim of this work was to understand mechanisms triggered by HPV associated tumor cells that control inflammation within the tumor microenvironment and also understand how these tumors may promote leukocytosis and myeloid cell recruitment. We opted to study cervical cancer derived cell lines, because this is the best HPV associated tumor studied until now. We chose experimental models where we could compare cell lines derived from tumors from the same anatomical site, but with different status regarding HPV. Our results clearly showed differences in inflammatory infiltrate, cytokine expression profile and systemic effects between HPV positive tumors and a HPV negative tumor. Our findings identify targets that may be used for therapy against HPV associated tumors, not only in the cervix, but also, probably, in other anatomical sites.

## Material and Methods

### Tumor models

Cervical cancer derived cell lines HeLa, SiHa and C33A cells were kindly donated by Prof. Luisa Lina Villa (ICESP, Sao Paulo, Brazil). HeLa is positive for HPV18, SiHa for HPV16 and C33A is negative for HPV, but has mutated p53 [28–30]. Cells were detached from the culture flasks by trypsin treatment, ressuspended in 10% Fetal Bovine Serum (FBS) RPMI, counted, washed twice with PBS^++^ (phosphate buffered saline supplemented with 1 mM CaCl_2_ and 0,5 mM MgCl_2_) and injected subcutaneously into RAG1^−/−^ mice (B6.129S7-Rag1^tm1Mom/^J, The Jackson Laboratory, Bar Harbor, Maine) or Nude mice (Nude NIH-III, Charles River Laboratories, Wilmington, MA). These mice were maintained in the Isogenic Mouse Facility at the Institute of Biomedical Sciences, with food and water *ad libidum*, 12 h cycles of light and dark and *spf* conditions. All animal experimentation procedures were approved by the Institutional Committee for Animal Use in Experimentation, under the protocol number 151/2010. Each mouse was injected with 5 × 10^6^ cells and evaluated every week until tumor detection and then every other day until tumors reached the maximum diameter of 7 mm. At this point, each mouse received an intraperitoneal injection of 1mg of bromodeoxyuridine (BrdU) (Sigma–Aldrich, St Louis, MO), and was anesthetized and euthanized 1 h later. We harvested the tumor, spleen, and bone marrow from each mouse.

### Tissue processing

We split the tumors in two fragments. One was frozen in Tissue-Tek OCT compound (Sakura-Finetek, Torrance, CA) and the other was finely minced and digested with 1 mg/ml Collagenase I and IV in MTH buffer (1x Hanks' buffered salt solution, 15 mM HEPES pH 7,4, 5% FBS, 0,5 U/ml DNAse I) at 37°C, under agitation of 1300 rpm, in a Thermomixer (Eppendorf, New York, NY). After digestion, cells were washed in MTH, counted, and stained for immunophenotyping.

Bone marrow and spleens were mechanically disrupted. Erythrocytes were eliminated by ACK lysis (0.15 M ammonium chloride, 10 mM potassium bicarbonate, 0.1 mM EDTA). Cells were counted and stained for immunophenotyping. BrdU was detected through intracellular staining after cells fixation, permeabilization and DNA fragmentation, using the BrdU detection kit (BD Biosciences, San Jose, CA). Alternatively, after fixation and permeabilization, we stained cells with DAPI for quantification of DNA content [31].

All stained cells were analyzed by flow cytometry in a FACSCalibur or FACSCanto II (BD Biosciences), where at least 30,000 events were acquired.

### Immunohistochemistry and histology

Frozen tumor fragments were sectioned into 5 μm sections. Cryo-sections were fixed in a mix of acetone and methanol (2:1 volumes) at room temperature for 5 min. Air-dried sections were hydrated by three PBS incubations, 2 min each, at room temperature. Endogenous peroxidase activity was quenched with 3% H_2_O_2_ for 10 min, and endogenous biotin was blocked with the Avidin/Biotin Blocking Kit (Vector Laboratories, Burlingame, CA). We also performed a blocking step with 5% FBS and 1 μg/ml FcBlock (BD Biosciences) for 30 min in PBS, prior to incubation of sections with primary antibody. Rat anti-CD45 antibody was diluted in 5% FBS in PBS and incubated on the tissue for 1 h. Sections were washed and the antibody was detected using the ABC Vectastain kit and DAB, 3,3′-diaminobenzidine (Vector Laboratories, Burlingame, CA). We performed counterstaining with Harrys Hematoxylin (VectorLaboratories) and Eosin (Sigma–Aldrich), dehydrated the tissue sections and mounted with Permount (Thermo Fisher Scientific, Waltham, MA) and a coverslip. Images were acquired with a BX61 Olympus microscope (Olympus Corporation, Tokyo, Japan).

Transversal 5 μm spleen cryo-sections, were fixed as described above, stained with Harrys Hematoxylin, washed, dehydrated, and mounted with Permount and a coverslip. Images were acquired with a BX61 Olympus microscope (Olympus Corporation).

### Cytokine expression profile and Western blotting

Cell lysates were prepared from HeLa, SiHa, and C33A cultures (in vitro) and from tumor cells sorted from tumors grown in mice (in vivo). Tumor single cell suspensions were obtained as described above, and CD45^−^ cells (tumor cells) were sorted from inflammatory CD45^+^ cells with magnetic beads conjugated with anti-CD45 antibody (Miltenyi Biotec, Bergisch Gladbach, Germany). Cells were lysed with reagents from the Proteome Cytokine Array Kit, according to the manufacturer's instructions (R&D Systems, Minneapolis, MN). We used 100 μg of protein for each array and ECL Chemiluminescence kit (GE Healthcare, Waukesha, WI) for detection of cytokine/antibody complexes. The resulting autoradiograms were scanned for densitometry.

For Western blottings, we used 25 μg of proteins from CD45^+^ and CD45^−^ cell lysates. Proteins were fractioned by SDS–Page and transferred to PVDF membranes (GE Healthcare). After blocking with 5% skim milk in 0,1% Tween 20/PBS, membranes were incubated with antibodies against anti-phosphorylated STAT3 and NFκB (p65) or anti-STAT3, NFκB and Akt (Cell Signaling Technology, Danvers, MA). Anti-tubulin was purchased from Sigma–Aldrich (St. Louis, MO). Secondary antibody was anti-rabbit from GE Healthcare, and detection was performed with ECL as described above.

### Statistical analyzes

We used both one-way ANOVA and *t*-test for our statistical analyzes. In both cases, differences between groups were considered significant if *P* < 0.05. *t*-Test was used to compare HPV positive and negative cells or tumors, and for comparisons between cells from tumor bearing mice and control mice.

## Results

### Tumor inflammatory infiltrate is different between HPV positive and negative tumors

**Figure 1:**
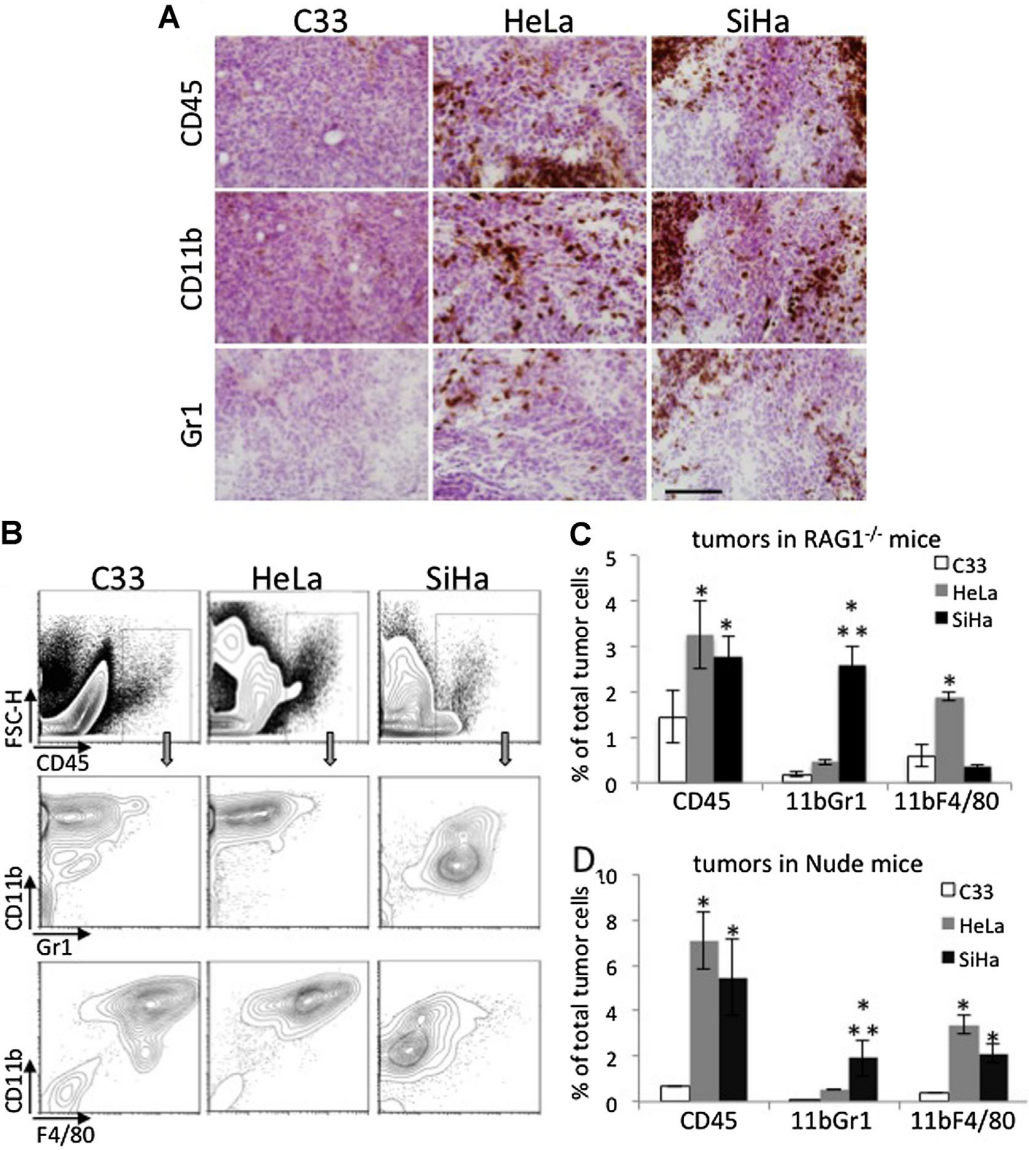
Leukocyte infiltrate in HeLa, SiHa, and C33A tumors. A: Immunohistochemistry from tumor sections stained with anti-CD45, anti-CD11b, and anti-Gr1. Tissue cryo-sections were incubated with the indicated antibodies, which were detected with the Vectastain Elite kit. Sections were counterstained with hematoxylin and eosin before mounting with Permount. Images were acquired in an Olympus BX61 microscope with 100× magnification. The bar indicates 100 μm. B–D: Flow cytometry analyzes of tumor cell suspensions. After harvesting, tumors were digested with Collagenase. Single cells suspensions were washed, aliquoted, and stained with antibodies against the indicated cell surface markers. In B, we show the flow cytometry results of a representative experiment. Upper panels display the CD45^+^ population, within which, all other analyzes were performed (arrows indicate the CD45^+^ population gate that contained the other populations). C and D. Average of cell population frequency in tumors in RAG1^−/−^ mice (C) and Nude mice (D). Each experimental group had, at least, four mice. * indicates significant differences in a population compared with the same in the C33A tumors, ** indicates significant differences between HeLa and SiHa tumors.

All tumor cell lines were capable of growing in both RAG1^−/−^ and Nude mice. The first difference we observed related to these tumors, was cell viability. At the end of the enzymatic dissociation, cells were washed and counted with Trypan blue to determine cell numbers and viability. Although tumors were processed simultaneously and under the same conditions, C33A tumor cell suspensions displayed significantly lower viability compared to SiHa and HeLa (57 ± 6.7%, 98 ± 3% and 92 ± 5.6%, respectively). We also observed, through immunohistochemistry and flow cytometry analyzes, that HeLa and SiHa tumors had significantly higher frequency of inflammatory cells than C33A in both RAG1^−/−^ and Nude mice (Fig. 1A and B, upper panels). Through immunohistochemistry, we detected both CD11b^+^ and Gr1^+^ cells in both HeLa and SiHa tumors (Fig. 1A, middle and lower panels). By flow cytometry, we observed that HeLa tumors had two fold more macrophages (CD45^+^CD11b^+^F4/80^+^) than CD11b^+^Gr1^+^ cells in RAG1^−/−^ mice and fourfold more in Nude mice (Fig. 1B–D). In SiHa tumors, we observed sixfold more CD11b^+^Gr1^+^ cells than macrophages in RAG1^−/−^ mice, and similar proportions of these populations in Nude mice (Fig. 1B–D). C33A tumors were mostly infiltrated by macrophages (Fig. 1B–D).

**Figure 2:**
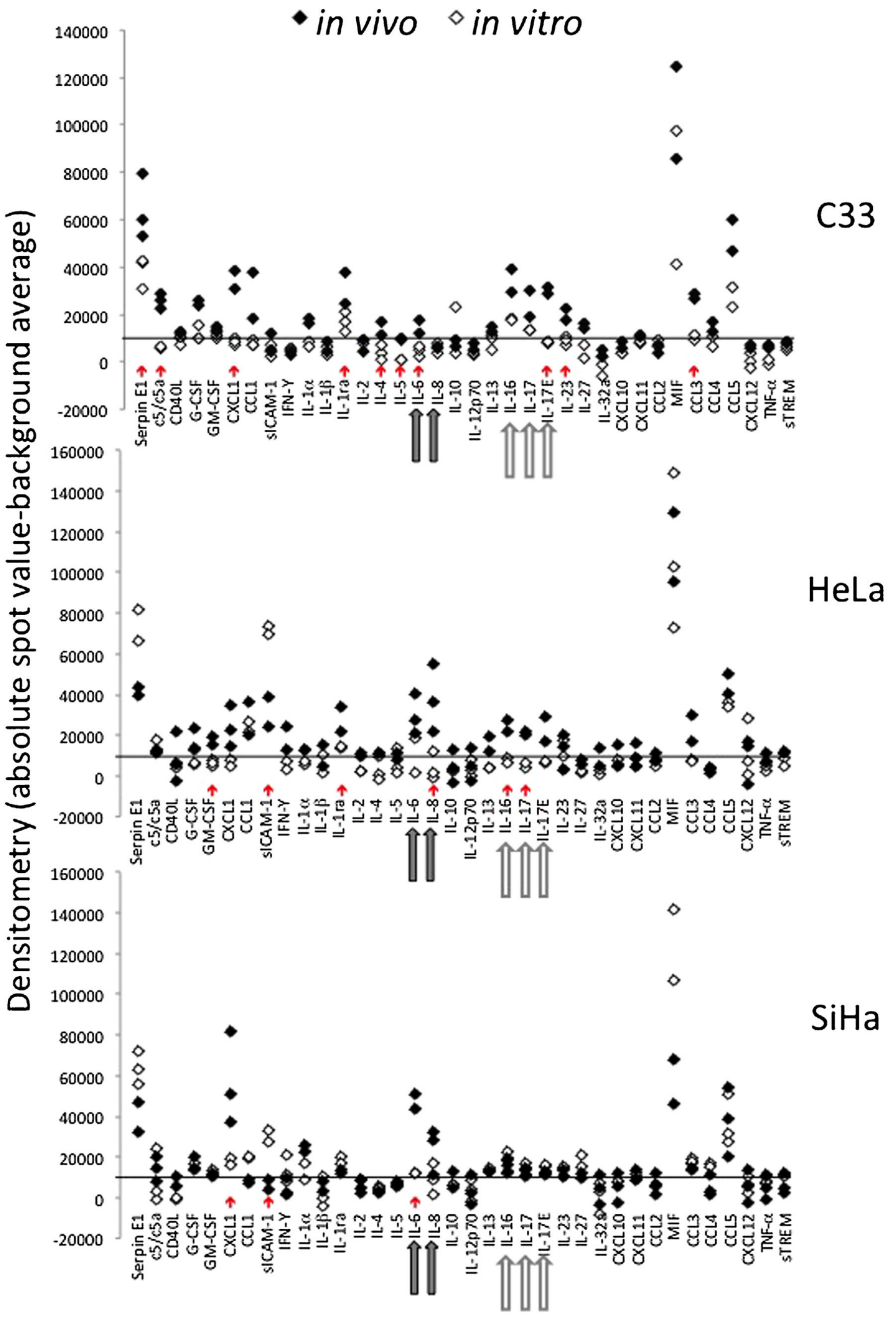
Cytokine expression profiles in in vitro and in vivo cervical cancer cell lines. Cell lysates were prepared from cells in culture (in vitro, black diamonds) or from cells sorted from tumors (in vivo, white diamonds). Sorted cells were obtained from tumors single cell suspensions eluted from magnetic columns after labeling with anti-CD45 magnetic beads. For all incubations, we used 100 μg of protein. Each of the lysates was incubated with membranes from the Human Cytokine Proteome Array kit, according to manufacturer's instructions. Cytokine/antibody complexes were detected using the ECL Chemiluminescence kit. Autoradiograms were scanned and densitometry data was used to generate the results. The graphs show absolute densitometry from each cytokine minus the average of six background spots with the same area of all other spots. The black lines in each graph represent the determined threshold of detection. Red arrows indicate significant differences between cytokine expression in a given cell line in vitro and in vivo. Gray arrows indicate cytokines with significant higher expression in HPV positive cell lines. White arrows indicate cytokines with significant higher expression in C33A. Three independent experiments are represented in this figure.

Cytokines and chemokines have an essential role in inflammatory cell recruitment, activation and phenotype. Therefore, we proceeded to investigate cytokine expression in tumor cells in vitro and in vivo. This experiment had two objectives, identify cytokines differentially expressed among the cervical cancer cell lines and observe the effect that tumor microenvironment may have on the tumor cells. It is important to highlight that to estimate cytokine expression from tumor cells in vivo, we sorted CD45^−^ cells from tumor cell suspensions immediately after tumor harvesting from mice. These preparations had average purity of 99.9%, therefore free of inflammatory cells. As observed in Figure 2, all cell lines displayed different cytokine expression profiles in vitro and in vivo (red arrows below each graph indicate significant differences between in vivo and in vitro cells). In general, we observed that the cytokine expression profiles of the cell lines in vitro were relatively homogeneous (Fig. 2, white markers). However, in vivo, HPV positive cell lines displayed significant differences in relation to C33A (Fig. 2, dark and light gray arrows under the abscissa axis). IL-6 and IL-8 (gray arrows) together with IL-16, IL-17, and IL-17E (white filled arrows) expression profiles differentiated HPV positive and negative tumor cells in vivo. HPV positive cell lines displayed significantly higher IL-6 and IL-8 expression than C33A, and the opposite was observed regarding IL-16, IL-17, and IL-17E. While there was no modulation of sICAM expression in C33A cells, both SiHa and HeLa cells downregulated sICAM expression in vivo. Although not significant, we observed that Serpin E1, the Plasminogen activator inhibitor-1, was upregulated in C33A tumors, while downregulated in SiHa and HeLa tumors. SiHa cells additionally expressed CXCL1 in higher levels than the other cell lines Finally, we observed relatively high expression levels of MIF and CCL5 in all experimental conditions.

**Figure 3:**
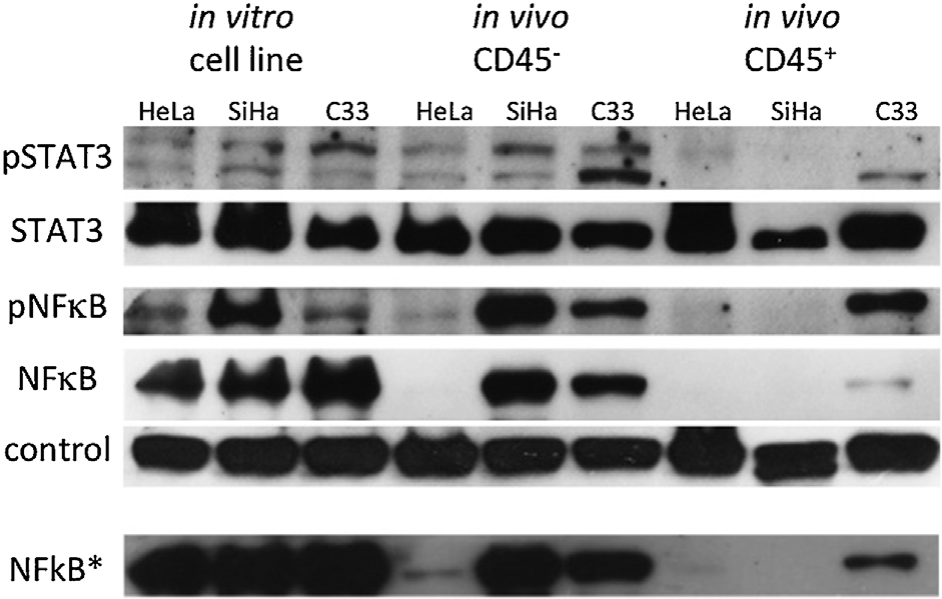
Signaling proteins expression in cervical cancer cell lines. Western blotting analyzes of 40 μg of proteins from cells in culture or from sorted CD45^−^ and CD45^+^ tumor cells. Sorted cells were obtained from tumors single cell suspensions eluted from magnetic columns after labeling with anti-CD45 magnetic beads. The membranes were first incubated with anti-phosphorylated proteins, as indicated in the left side of each autoradiogram. After striping in acidic glycine, Tris solution, membranes were incubated with antibodies that recognize the indicated proteins independently of post-translational modifications. This is one representative experiment of two independent ones. Control corresponds to Akt expression, which we have observed to be constitutive among all our expression conditions. The last reaction depicted, NFκB* is a longer exposure of the same autoradiogram shown for NFκB.

Tumors are typically chronic diseases. Therefore, tumor cells generate persistent signals that have autocrine, paracrine, or endocrine effects. Long-term exposure to stimuli can downregulate signaling pathways [32]. In our model, tumors take up to two months to reach the maximum acceptable diameter, which means that the cytokines expressed by the different tumors can stimulate cells from the host, locally and systemically, in a chronic manner. Given the differential cytokine expression profiles between HPV positive and negative cell lines in vivo, we asked whether we would find any differences in signaling pathways in tumor cells and inflammatory infiltrates. In Figure 3, we show the results of STAT3 and NFκB protein expression. Both are key proteins in signaling pathways involved in inflammatory responses and cancer [33,34]. In these experiments, we used cell lysates from tumor cells in vitro, CD45^−^ sorted tumor cells (in vivo) and from sorted CD45^+^ leukocyte infiltrate. We did not observe significant differences in total STAT3 and NFκB protein expression among tumor cells either in vitro or in vivo, except for the low NFκB expression in HeLa cells in vivo (a longer exposed autoradiogram shows a band correspondent to NFκB in HeLa cells). We noticed alterations regarding the phosphorylated isoforms of our proteins of interest, mainly NFκB. First, we observed that in vivo SiHa cells displayed higher levels of phosphorylated NFκB than the other cell lines. More striking, however, was the absence of phosphorylated NFκB expression in the inflammatory infiltrate of HeLa and SiHa tumors. Phosphorylated STAT3 expression was very low in the inflammatory infiltrates of HeLa and SiHa tumors compared to the expression in the leukocytes present in C33A tumors.

### 2. HPV positive cell lines induce myeloid cell proliferation and accumulation in lymphoid organs

**Figure 4:**
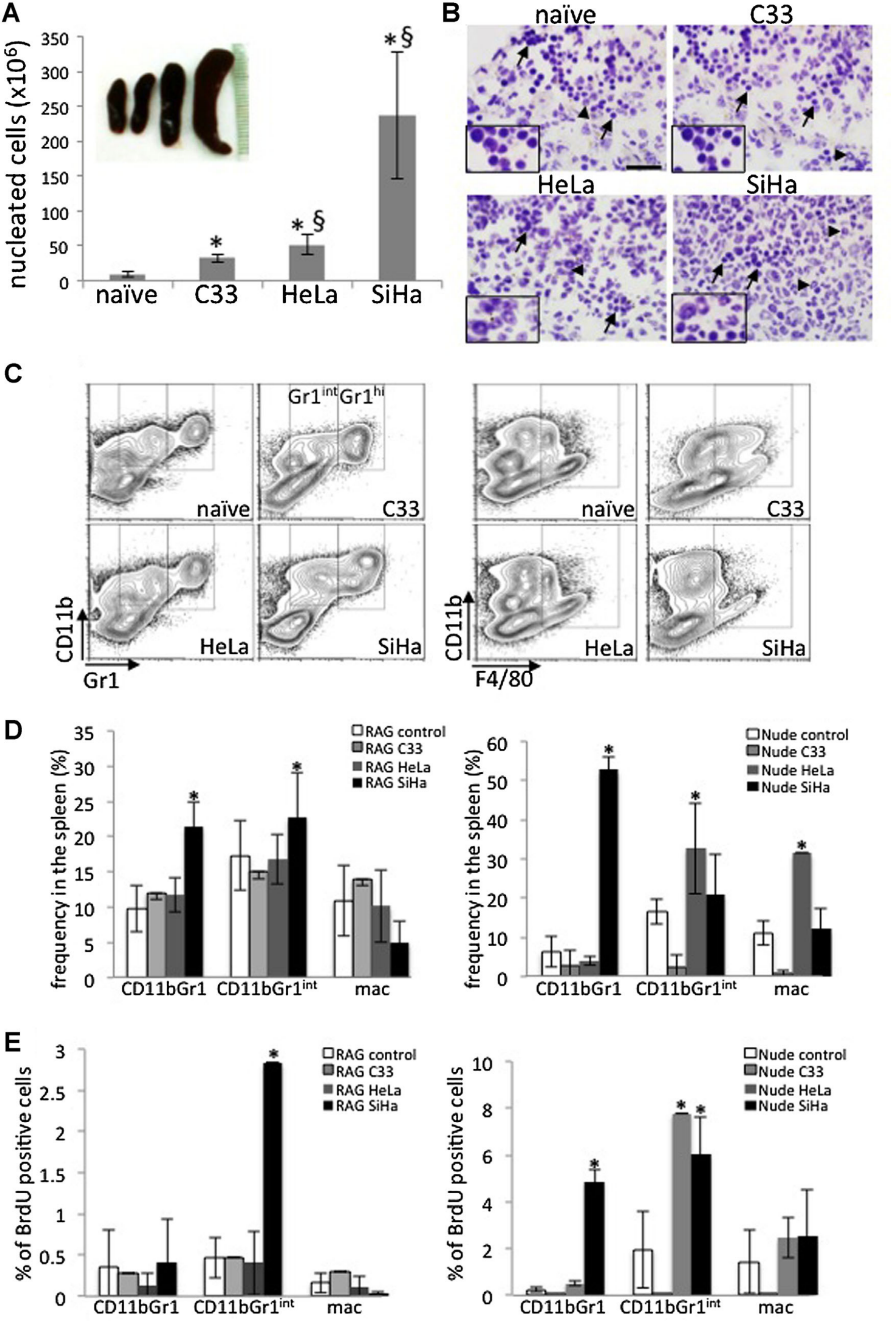
HPV positive tumors induce accumulation of myeloid cells in the spleen of mice. A: Number of nucleated cells in the spleen of control mice (naïve) or mice bearing C33A, HeLa, and SiHa tumors, respectively. After spleen dissociation and red cell lysis, nucleated cells were counted in Neubauer chambers to obtain the number of nucleate cells/spleen. All groups had significantly more cells than control naïve mice (*), HeLa and SiHa tumor bearing mice had significantly more cells than C33A tumor bearing mice (§). The inset over the graph shows a representative photograph of the spleens of tumor bearing mice, in the same order they are represented in the graph. B: Cell morphology in the spleens of control mice (naïve) and tumor bearing RAG1^−/−^ mice (lineages indicated above each image). Frozen and fixed spleen sections were stained with hematoxylin prior to mounting the slides. 400× magnification images were acquired with an Olympus BX61 microscope. The insets show nuclei details under 1000× magnification. Scale bar indicates 100 μm. Arrows indicate mononuclear cells and arrowheads indicate cells with doughnut shaped nuclei. C. Representative experiment of flow cytometry analyzes of myeloid CD11b^+^Gr1^+^splenocytes (left) and CD11b^+^F4/80^+^splenocytes (right) from RAG1^−/−^ mice. In the right panels, gates indicate the CD11b + Gr1int and Gr1+ populations as indicated. In the right panels, gates indicate the macrophage populations, independently of F4/80 staining intensity. D. Quantification of splenocyte populations analyzed by flow cytometry, within the nucleated population, as represented in C (right side splenocytes from RAG1^−/−^ mice, left side from Nude mice). E: The graphs in the right side show the percentage of BrdU incorporating cells within each specific population in C (right side splenocytes from RAG1^−/−^ mice, left side from Nude mice). * indicates significant differences in comparison to the control (naïve) group. Mac corresponds to CD11b^+^F4/80^+^, which we assume are macrophages.

**Figure 5:**
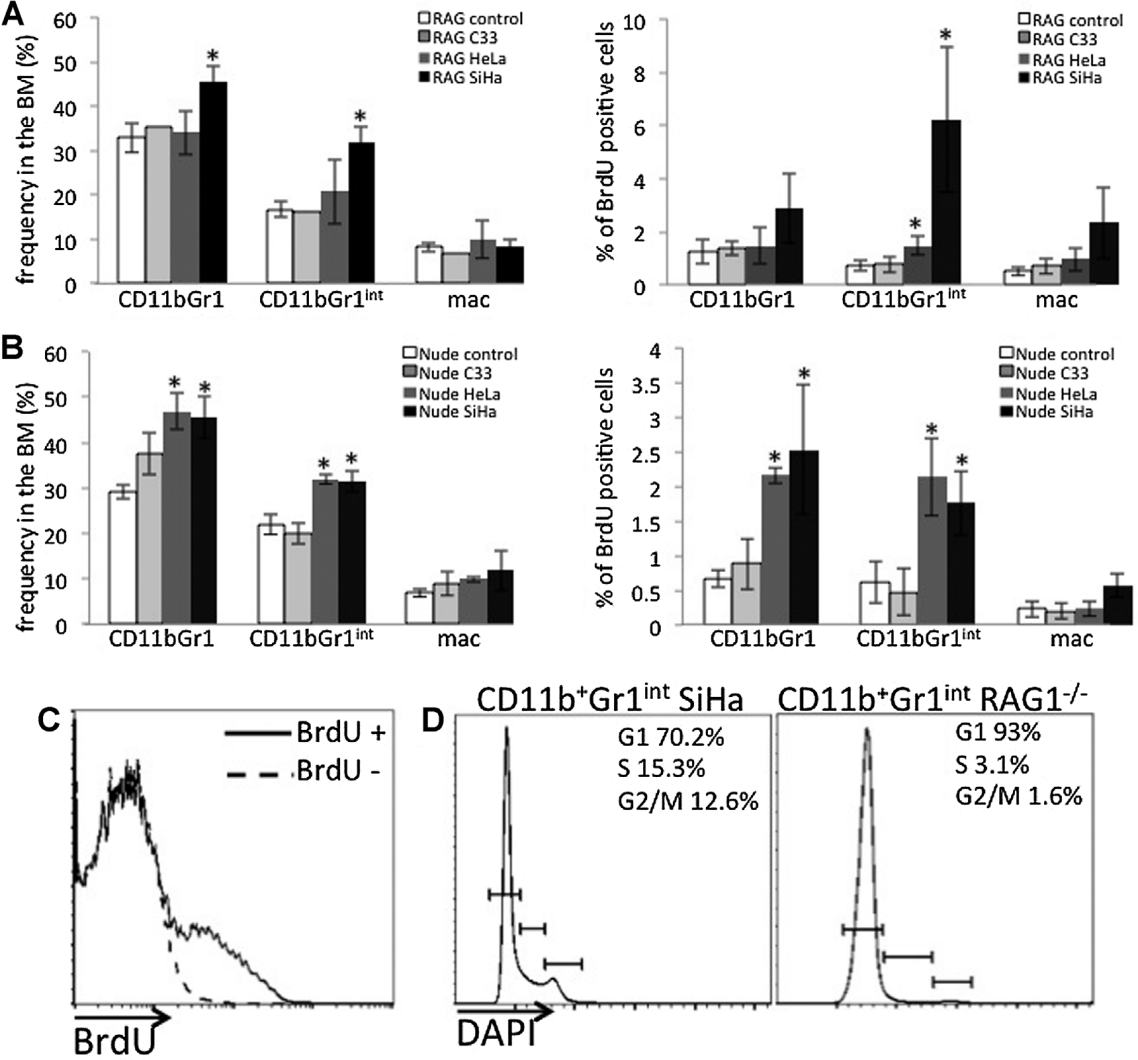
HPV positive tumors induce accumulation of myeloid cells in the bone marrow of mice. Bone marrow single cell suspensions from RAG1^−/−^ (A) and Nude (B) mice were stained with antibodies against the indicated antigens and analyzed by flow cytometry. CD11b^+^Gr1^+^ population was split in Gr1int and Gr1^+^ for our analysis. The graphs in the left side show the frequency of each population within the nucleated cell population in control, C33A (C33), HeLa and SiHa tumor bearing mice. The graphs in the right side show the percentage of BrdU incorporating cells within each specific population. * indicates significant differences in comparison to the control group. Mac corresponds to CD11b^+^F4/80^+^, which we assume are macrophages. C: Splenocytes from mice injected or not with BrdU (BrdU +, solid line and BrdU-, dashed line, respectively) were processed the same way as cells in all other experiments and incubated with anti-BrdU in the same exact conditions. This experiment demonstrates that the antibody anti-BrdU is specific and our results are not a staining artifact. D. Splenocytes from control RAG1^−/−^ mice (RAG1^−/−^) or RAG1^−/−^ mice with SiHa tumor (SiHa) were stained with anti-CD11b and anti-Gr1, as described before. Cells were then fixed, permeabilized and incubated with 10 μg/ml DAPI for 30 min, washed and ressuspended in the same DAPI concentration right before acquisition [22]. The histograms represent frequency of cells in each cell cycle phase, G1, S and G2/M, as specified in the right side of each graph, within the CD11b^+^Gr1^int^ population.

One of the first observations we made when we evaluated mice with tumors was that mice with HeLa and, more dramatically, with SiHa tumors had spleen hyperplasia compared to C33A and control mice (Fig. 4A). This effect was only observed in RAG1^−/−^ mice, probably because their spleens are empty of lymphocytes, therefore easier to observe alterations in myeloid populations. Interestingly, there were mainly monocytic cells in the spleen of naive and C33A injected mice (Fig. 4B). In mice with HeLa tumors, we observed a mix of monocytic cells and cells with nuclei in the shape of a doughnut. In SiHa injected mice, we found mainly cells with doughnut shaped nuclei (Fig. 4B). We analyzed these spleens single cell suspensions by flow cytometry with focus on macrophages CD11b^+^F4/80^+^ cells and myeloid cells CD11b^+^Gr1^+^ and CD11b^+^Gr1^int^. We split the analyzes of CD11b^+^Gr1^+^ cells in Gr1^+^ and Gr1^int^ populations because looking at CD11b versus Gr1 dot plots, we could actually see different populations. Data in the literature suggests that CD11b^+^Gr1^int^ cells may be myeloid suppressor derived cells, while CD11b^+^Gr1^+^ would be neutrophils or other granulocytes [35]. As shown in Figures 4C and D, splenocytes from RAG1^−/−^ mice with SiHa tumors displayed significantly higher frequencies of CD11b^+^Gr1^+^ and CD11b^+^Gr1^int^ cells than splenocytes from control naïve mice. In Nude mice (Fig. 4D, right panel), we observed similar results when mice were injected with SiHa cells, and also observed an increase in CD11b^+^Gr1^int^ and macrophages in mice injected with HeLa cells. We did not observe differences in the frequency of the investigated populations in the spleen of C33A tumor bearing mice. We used in vivo metabolic incorporation of BrdU to estimate cell proliferation in tumor bearing and naïve mice. In Figure 4E, we show that in RAG1^−/−^ mice, the percentage of CD11b^+^Gr1^+^cells incorporating BrdU was significantly higher in SiHa tumor bearing mice than in control mice (5.6-fold increase). In Nude tumor bearing mice, we observed an increase in the numbers of BrdU incorporating CD11b^+^Gr1^+^ and CD11b^+^Gr1^int^ cells in mice with SiHa tumors (20-fold and 3-fold increase, respectively) and CD11b^+^Gr1^int^ cells in mice with HeLa tumors (3.95-fold increase). As controls, we performed the same assay in mice without BrdU injections, where we observed that our anti-BrdU antibody is specific (Fig. 5C). We also did cell cycle analysis with DAPI stained cells, and observed that populations that displayed an increase in BrdU incorporation also had higher percentages of cells in S and G2/M phase (Fig. 5D). These results corroborate the data showing that in SiHa tumors CD11b^+^Gr1^+^ cells are more frequent, while HeLa tumors have mixed populations.

We also investigate myeloid cell frequency and proliferation in the bone marrow of tumor bearing mice. As in the spleen, we did not observe an increase in the investigated populations frequency or proliferation, in the bone marrow of C33A tumor bearing mice. We did observe an increase in CD11b^+^Gr1^+^ and CD11^+^Gr1^int^ cells in SiHa bearing RAG1^−/−^ mice, and of CD11b^+^Gr1^int^ in SiHa bearing Nude mice (Fig. 5A). Interestingly we observed that in both SiHa and HeLa tumor bearing mice, there was an increase in BrdU incorporation in CD11b^+^Gr1^+^ and CD11b^+^Gr1^int^ cells, in both mouse strains (Fig. 5B). We did not observe any significant increase in macrophage proliferation in the bone marrow or spleen of tumor bearing mice, indicating that any increase in this population may be derived from differentiation from CD11b^+^Gr1^+/int^ cells.

## Discussion

This manuscript shows the effects of HPV associated tumors from signaling in the populations present in the tumor microenvironment to proliferation and frequency of cells in lymphoid organs. Our results show that local inflammation and systemic effects upon myeloid cells are different between HPV positive and negative cervical cancer derived cell lines. Interestingly, SiHa and HeLa are derived from a squamous carcinoma and an adenocarcinoma, respectively, indicating that HPV overcomes any differences these cells might have, related to inflammation, in spite of cell origin. Pyeon and collaborators reached similar conclusions comparing head and neck tumors positive or negative for HPV with cervical cancer derived cell lines, in a study focused on the effects of HPV oncoproteins on cell cycle control [36].

**Figure 6:**
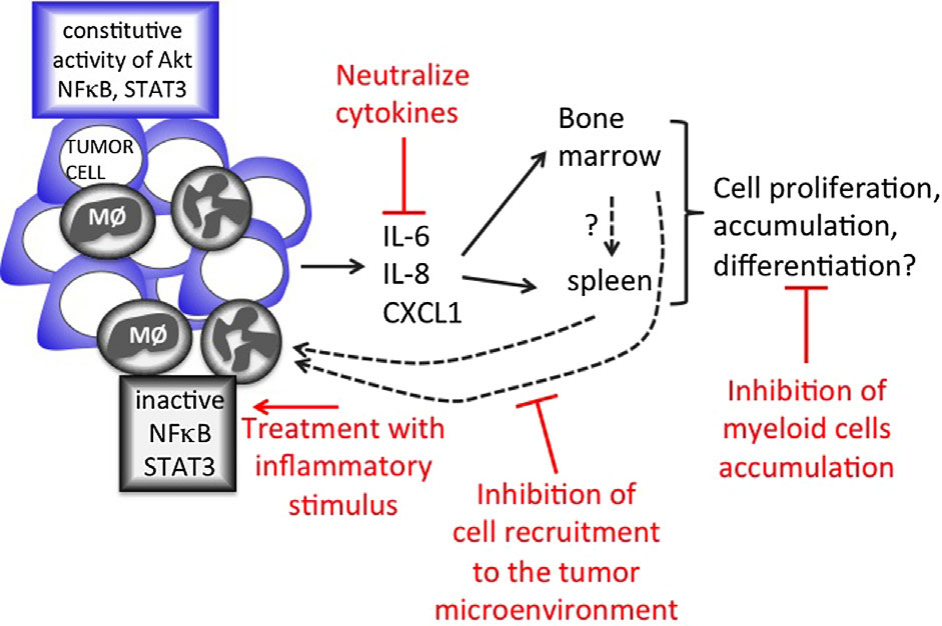
Schematic diagram of local and systemic tumor effects. In HPV associated tumors, we observed the presence of macrophages (MØ) and CD11b^+^Gr1^+^, here represented as polymorphonuclear cells. HPV associated tumor cells express IL-6, IL-8, and CXCL1, which may have systemic effects, promoting cell proliferation, accumulation, and possibly influence differentiation of myeloid cells in the bone marrow and spleen. We have not tested whether myeloid cells migrate from the bone marrow to the spleen, but due to the correlation of the type of cells expanding in the lymphoid organs and infiltrating the tumors, we believe these cells migrate to the tumor, where they have a suppressor like phenotype with low phosphorylated STAT3 expression, and no phosphorylated NFκB expression. In red, we pointed out possible mechanisms to inhibit tumor growth by inhibiting cell proliferation and recruitment, neutralizing cytokines or generating inflammatory signals that could activate infiltrating leukocytes.

Except for C33A, we observed a parallel between the type of cell populations expanding in lymphoid tissues and the populations infiltrating tumors. Our hypothesis is that tumors create a vicious circle, where molecules generated and secreted by the tumor cells induce expansion and recruitment of myeloid cells to the tumor microenvironment (Fig. 6). Once in the tumor, these cells contribute to the tumor microenvironment, which modulates cytokine expression in tumor cells, feeding back the cycle. Interestingly, although the tumor microenvironment modulated cytokine expression in tumor cells, there was no significant difference in the activation of the evaluated signaling pathways in these cells. It is possible that other signaling pathways may have been modulated in the tumor cells, but this remains to be investigated.

One of the cytokines possibly involved in myeloid cell accumulation is IL-6. IL-6 has been described as capable of inducing myeloid cell proliferation and splenomegaly, as well as inducing suppressor phenotype in these cells in mice [37,38]. In vitro experiments have shown that IL-6, together with PGE2, induce suppressor phenotype on monocyte derived dendritic cells [39]. Moreover, IL-6 expression has been associated with disease severity in cervical, head and neck and breast cancer patients [40–42]. While, we believe that chronic IL-6 expression may be responsible for STAT3 and NFκB downregulation in tumor associated inflammatory cells; its systemic effect may be stimulatory, due to lower concentration in the body compared to the intratumoral concentration. It is important to mention that while we measured only proliferation of cells in lymphoid tissues, it is possible cell survival may also be impacted by tumors.

Among the cytokines upregulated by the tumor microenvironment, we found IL-8 and CXCL1, which display redundant activities, chemoattracting neutrophils, inducing angiogenesis, cell survival and tumor evasion, including in patients with cervical cancer [43–46]. Data in the literature also indicates that CXCL1can recruit MDSC (myeloid derived suppressor cells) to breast tumors [47]. It is of notice that SiHa tumors, which had the most robust effect in accumulation of myeloid cells in lymphoid tissues and recruitment to the tumor, displayed higher expression of IL-8 and significantly higher expression of CXCL1 in vivo than in vitro, indicating a potential role for these chemokines in the mechanism of action of SiHa tumors. At this point, we do not know if CD11b^+^Gr1^+^ cells observed in our experimental models are MDSC or neutrophils. In mice, MDSC may have doughnut shaped nuclei [48], indicating that this population is present in our HPV positive tumor hosts. However, specific assays should be performed to confirm this hypothesis.

In C33A cells isolated from tumors, we observed increased expression of IL-16 and IL-17, cytokines typically associated with T cell activity. Recently, several groups have shown that IL-16 display pro-tumoral activity in cutaneous T cell lymphoma, multiple myeloma and breast cancer [49,50]. Although through different mechanisms, IL-16 induced proliferation of lymphocytes in T cell lymphomas and multiple myeloma and recruitment of pro-tumoral macrophages to breast tumors. IL-17 has been associated to different types of tumors, mainly due to the role of Th17 CD4 T cells or innate lymphoid cells in chronic inflammation [51–53]. Although there are no lymphocytes in our model, our data shows that tumor cells themselves express IL-16 and IL-17. There is evidence of IL-17C expression in epithelial cells [54], but, as far as we could find, there is no evidence of epithelial cells expressing IL-16 in the literature.

Our laboratory has been investigating the status of signaling pathways in experimental models and cervical cancer biopsies. The results regarding the inactivity of NFκB in the inflammatory infiltrate of SiHa and HeLa tumors are consistent with what we have been observing in an isogenic HPV16 associated tumor model and in cervical cancer biopsies (data not shown). At this moment, we believe that inactivity or impaired activity of NFκB may be a mechanism that renders inflammatory cells less likely to respond and present antigens from tumors, therefore consisting of used by HPV associated tumors. It will be of interest to design methods to activate NFκB in vivo and test this hypothesis.

Together, our results points to therapeutic possibilities. We have shown before that depletion of tumor macrophages from a HPV16 positive tumor model inhibits tumor growth [23]. Figure 6 shows a diagram of the interaction between the tumor microenvironment and leukocytes in the host's lymphoid organs. It also shows targets that may be use for therapy in cervical cancer, and possibly in HPV positive tumors in the oropharynx and other ano-genital areas.

## SUPPORTING INFORMATION

**Figure S1.** Signaling proteins expression in cervical cancer cell lines.

## References

[1] Bosch FX, Broker TR, Forman D, Moscicki AB, Gillison ML, Doorbar J, Stern PL, Stanley M, Arbyn M, Poljak M (2013). Comprehensive control of hpv infections and related diseases. Vaccine.

[2] Walboomers JM, Jacobs MV, Manos MM, Bosch FX, Kummer JA, Shah KV, Snijders PJ, Peto J, Meijer CJ, Muñoz N (1999). Human papillomavirus is a necessary cause of invasive cervical cancer worldwide. J. Pathol.

[3] Pirami L, Giachè V, Becciolini A (1997). Analysis of HPV16, 18, 31, and 35 DNA in pre-invasive and invasive lesions of the uterine cervix. J. Clin. Pathol.

[4] Cullen AP, Reid R, Campion M, Lorincz AT (1991). Analysis of the physical state of different human papillomavirus DNAs in intraepithelial and invasive cervical neoplasm. J. Virol.

[5] Duensing S, Münger K (2003). Centrosome abnormalities and genomic instability induced by human papillomavirus oncoproteins. Prog. Cell Cycle Res.

[6] Johung K, Goodwin EC, DiMaio D (2007). Human papillomavirus E7 repression in cervical carcinoma cells initiates a transcriptional cascade driven by the retinoblastoma family, resulting in senescence. J. Virol.

[7] Fujii T, Saito M, Iwasaki E, Ochiya T, Takei Y, Hayashi S, Ono A, Hirao N, Nakamura M, Kubushiro K (2006). Intratumor injection of small interfering RNA-targeting human papillomavirus 18 E6 and E7 successfully inhibits the growth of cervical cancer. Int. J. Oncol.

[8] Tindle RW (2002). Immune evasion in human papillomavirus-associated cervical cancer. Nat. Rev. Cancer.

[9] de Gruijl TD, Bontkes HJ, Walboomers JM, Stukart MJ, Doekhie FS, Remmink AJ, Helmerhorst TJ, Verheijen RH, Duggan-Keen MF, Stern PL (1998). Differential T helper cell responses to human papillomavirus type 16 E7 related to viral clearance or persistence in patients with cervical neoplasia: a longitudinal study. Cancer Res.

[10] de Jong A, van der Burg SH, Kwappenberg KM, van der Hulst JM, Franken KL, Geluk A, van Meijgaarden KE, Drijfhout JW, Kenter G, Vermeij P (2002). Frequent detection of human papillomavirus 16 E2-specific T-helper immunity in healthy subjects. Cancer Res.

[11] Barashi N, Weiss ID, Wald O, Wald H, Beider K, Abraham M, Klein S, Goldenberg D, Axelrod J, Pikarsky E (2013). Inflammation induced hepatocellular carcinoma is dependent on CCR5. Hepatology.

[12] Guerra C, Collado M, Navas C, Schuhmacher AJ, Hernández-Porras I, Cañamero M, Rodriguez-Justo M, Serrano M, Barbacid M (2011). Pancreatitis-induced inflammation contributes to pancreatic cancer by inhibiting oncogene-induced senescence. Cancer Cell.

[13] Pollard JW (2008). Macrophages define the invasive microenvironment in breast cancer. J. Leukoc. Biol.

[14] Candido J, Hagemann T (2013). Cancer-related inflammation. J. Clin. Immunol.

[15] Balkwill FR, Mantovani A (2012). Cancer-related inflammation: common themes and therapeutic opportunities. Semin. Cancer Biol.

[16] Williams VM, Filippova M, Soto U, Duerksen-Hughes PJ (2011). HPV-DNA integration and carcinogenesis: putative roles for inflammation and oxidative stress. Fut. Virol.

[17] Feng Q, Wei H, Morihara J, Stern J, Yu M, Kiviat N, Hellstrom I, Hellstrom KE (2012). Th2 type inflammation promotes the gradual progression of HPV-infected cervical cells to cervical carcinoma. Gynecol. Oncol.

[18] Trimble C, Clark R, Hanson N, Tassello J, Frosina D, Teague J, Jiang J, Barat N, Kos F, Thoburn C (2010). Cervical mucosal CD8 T cells are more predictive of HPV lesion regression than systemic HPV-specific response. J. Immunol.

[19] Piersma SJ, Jordanova ES, van Poelgeest MI, Kwappenberg KM, van der Hulst JM, Drijfhout JW, Melief CJ, Kenter GG, Fleuren GJ, Offringa R (2007). High number of intraepithelial CD8+ tumor-infiltrating lymphocytes is associated with the absence of lymph node metastases in patients with large early-stage cervical cancer. Cancer Res.

[20] Kobayashi A, Weinberg V, Darragh T, Smith-McCune K (2008). Evolving immunosuppressive microenvironment during human cervical carcinogenesis. Mucosal Immunol.

[21] Mazibrada J, Rittà M, Mondini M, De Andrea M, Azzimonti B, Borgogna C, Ciotti M, Orlando A, Surico N, Chiusa L (2008). Interaction between inflammation and angiogenesis during different stages of cervical carcinogenesis. Gynecol. Oncol.

[22] Pahler JC, Tazzyman S, Erez N, Chen YY, Murdoch C, Nozawa H, Lewis CE, Hanahan D (2008). Plasticity in tumor-promoting inflammation: impairment of macrophage recruitment evokes a compensatory neutrophil response. Neoplasia.

[23] Lepique AP, Daghastanli KR, Cuccovia IM, Villa LL (2009). HPV16 tumor associated macrophages suppress antitumor T cell responses. Clin. Cancer Res.

[24] Garcia-Arias A, Cetina L, Candelaria M, Robles E, Dueñas-González A (2007). The prognostic significance of leukocytosis in cervical cancer. Int. J. Gynecol. Cancer.

[25] Mabuchi S, Matsumoto Y, Hamasaki T, Kawano M, Hisamatsu T, Mutch DG, Kimura T (2012). Elevated white blood cell count at the time of recurrence diagnosis is an indicator of short survival in patients with recurrent cervical cancer. Int. J. Gynecol. Cancer.

[26] Gabrilovich DI, Velders MP, Sotomayor EM, Kast WM (2001). Mechanism of immune dysfunction in cancer mediated by immature Gr-1+ myeloid cells. J. Immunol.

[27] Bolpetti A, Silva JS, Villa LL, Lepique AP (2010). Interleukin-10 production by tumor infiltrating macrophages plays a role in human papillomavirus 16 tumor growth. BMC Immunol.

[28] Leone V, Hsu TC, Pomerat CM Cytological studies on HeLa, a strain of human cervical carcinoma.II. 1955. On rotatory movements of the nuclei. Zeitschrift fur Zellforschung und mikroskopische Anatomie.

[29] Friedl F, Kimura I, Osato T, Ito Y (1970). Studies on a new human cell line (SiHa) derived from carcinoma of uterus. I. Its establishment and morphology. Proctol. Soc. Exp. Biol. Med.

[30] Crook T, Wrede D, Vousden KH (1991). p53 point mutation in HPV negative human cervical carcinoma cell lines. Oncogene.

[31] Gordon KM, Duckett L, Daul B, Petrie HT (2003). A simple method for detecting up to five immunofluorescent parameters together with DNA staining for cell cycle or viability on a benchtop flow cytometer. J. Immunol. Methods.

[32] Baniyash M (2004). TCR zeta-chain downregulation: curtailing an excessive inflammatory immune response. Nat. Rev. Immunol.

[33] He G, Yu G, Temkin V, Ogata H, Kuntzen C, Sakurai T, Sieghart W, Peck-Radosavljevic W, Leffert HL, Karin M (2010). Hepatocyte IKKβ/NF-κB inhibits tumor promotion and progression by preventing oxidative stress driven STAT3 activation. Cancer Cell.

[34] Alberti C, Pinciroli P, Valeri B, Ferri R, Ditto A, Umezawa K, Sensi M, Canevari S, Tomassetti A (2012). Ligand-dependent EGFR activation induces the co-expression of IL-6 and PAI-1 via the NFkB pathway in advanced-stage epithelial ovarian cancer. Oncogene.

[35] Youn J, Collazo M, Shalova IN, Biswas SK, Gabrilovich DI (2012). Characterization of the nature of granulocytic myeloid-derived suppressor cells in tumor-bearing mice. J. Leukoc. Biol.

[36] Pyeon D, Newton MA, Lambert PF, den Boon JA, Sengupta S, Marsit CJ, Woodworth CD, Connor JP, Haugen TH, Smith EM (2007). Fundamental differences in cell cycle deregulation in human papillomavirus-positive and human papillomavirus-negative head/neck and cervical cancers. Cancer Res.

[37] Ruscetti FW (1994). Hematologic effects of interleukin-1 and interleukin-6. Curr. Opin. Hematol.

[38] Jenkins BJ, Roberts AW, Greenhill CJ, Najdovska M, Lundgren-May T, Robb L, Grail D, Ernst M (2007). Pathologic consequences of STAT3 hyperactivation by IL-6 and IL-11 during hematopoiesis and lymphopoiesis. Blood.

[39] Heusinkveld M, de Vos van Steenwijk PJ, Goedemans R, Ramwadhdoebe TH, Gorter A, Welters MJ, van Hall T, van der Burg SH (2011). M2 macrophages induced by prostaglandin E2 and IL-6 from cervical carcinoma are switched to activated M1 macrophages by CD4+ Th1 cells. J. Immunol.

[40] Tjiong MY, van der Vange N, ten Kate FJ, Tjong-A-Hung SP, terSchegget J, Burger MP, Out TA (1999). Increased IL-6 and IL-8 levels in cervicovaginal secretions of patients with cervical cancer. Gynecol. Oncol.

[41] Ibrahim SA, Hassan H, Vilardo L, Kumar SK, Kumar AV, Kelsch R, Schneider C, Kiesel L, Eich HT, Zucchi I (2013). Syndecan-1 (CD138) modulates triple-negative breast cancer stem cell properties via regulation of LRP-6 and IL-6-mediated STAT3 signaling. PLoS ONE.

[42] Duffy SA1, Taylor JM, Terrell JE, Islam M, Li Y, Fowler KE, Wolf GT, Teknos TN (2008). Interleukin-6 predicts recurrence and survival among head and neck cancer patients. Cancer.

[43] Gouwy M, Struyf S, Noppen S, Schutyser E, Springael JY, Parmentier M, Proost P, Van Damme J (2008). Synergy between coproduced CC and CXC chemokines in monocyte chemotaxis through receptor-mediated events. Mol. Pharmacol.

[44] Waugh DJ, Wilson C (2008). The interleukin-8 pathway in cancer. Clin. Cancer Res.

[45] Vandercappellen J, Van Damme J, Struyf S (2008). The role of CXC chemokines and their receptors in cancer. Cancer Lett.

[46] Wu S, Shang H, Cui L, Zhang Z, Zhang Y, Li Y, Wu J, Li RK, Xie J (2013). Targeted blockade of interleukin-8 abrogates its promotion of cervical cancer growth and metastasis. Mol. Cell. Biochem.

[47] Acharyya S, Oskarsson T, Vanharanta S, Malladi S, Kim J, Morris PG, Manova-Todorova K, Leversha M, Hogg N, Seshan VE (2012). A CXCL1 paracrine network links cancer chemoresistance and metastasis. Cell.

[48] Van Ginderachter JA, Meerschaut S, Liu Y, Brys L, De Groeve K, Hassanzadeh Ghassabeh G, Raes G, De Baetselier P (2006). Peroxisome proliferator-activated receptor gamma (PPARgamma) ligands reverse CTL suppression by alternatively activated (M2) macrophages in cancer. Blood.

[49] Richmond J, Tuzova M, Cruikshank W, Center D (2014). Regulation of cellular processes by interleukin-16 in homeostasis and cancer. J. Cell. Physiol.

[50] Mahindra A, Anderson KC (2012). Role of interleukin 16 in multiple myeloma pathogenesis: a potential novel therapeutic target. J. Natl. Cancer Inst.

[51] Petanidis S, Anestakis D, Argyraki M, Hadzopoulou-Cladaras M, Salifoglou A (2013). Differential expression of IL-17, 22 and 23 in the progression of colorectal cancer in patients with K-ras mutation: Ras signal inhibition and crosstalk with GM-CSF and IFN-γ. PLoS ONE.

[52] Sundrud MS, Trivigno C (2013). Identity crisis of Th17 cells: Many forms, many functions, many questions. Semin. Immunol.

[53] Fuchs A, Colonna M (2013). Innate lymphoid cells in homeostasis, infection, chronic inflammation and tumors of the gastrointestinal tract. Curr. Opin. Gastroenterol.

[54] Song X, Gao H, Lin Y, Yao Y, Zhu S, Wang J, Liu Y, Yao X, Meng G, Shen N (2014). Alterations in the microbiota drive interleukin-17C production from intestinal epithelial cells to promote tumorigenesis. Immunity.

