# Supplementary figures and images for: HPV associated tumor cells control tumor microenvironment and leukocytosis in experimental models
Source: Immun Inflamm Dis. 2014 May 18;2(2):63–75. doi: 10.1002/iid3.21 (PMC4217549; doi:10.1002/iid3.21)

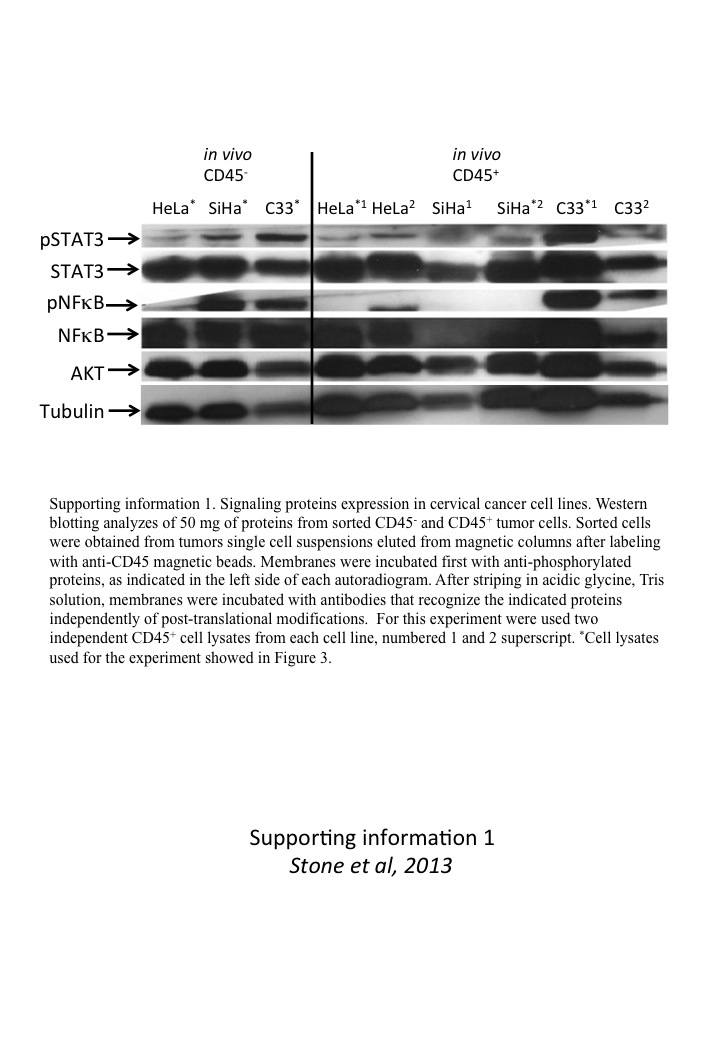

Supplement: Supplementary file 1 — Figure S1. Signaling proteins expression in cervical cancer cell lines. [file iid30002-0063-SD1.jpg]
